# Usage Pattern of Carbamazepine and Associated Severe Cutaneous Adverse Reactions in Singapore Following Implementation of HLA-B*15:02 Genotyping as Standard-of-Care

**DOI:** 10.3389/fphar.2020.00527

**Published:** 2020-05-07

**Authors:** Cynthia Sung, Liesbet Tan, Michael Limenta, Ganga Ganesan, Dorothy Toh, Cheng Leng Chan

**Affiliations:** ^1^Vigilance and Compliance Branch, Health Products Regulation Group, Health Sciences Authority, Singapore, Singapore; ^2^Health Services & Systems Research Programme, Duke-NUS Medical School, Singapore, Singapore; ^3^Policy Research and Evaluation Division, Ministry of Health, Singapore, Singapore

**Keywords:** HLA-B*15:02, carbamazepine, levetiracetam, Steven–Johnson syndrome, toxic epidermal necrolysis (Stevens–Johnson syndrome/toxic epidermal necrolysis), serious cutaneous skin reactions

## Abstract

In April 2013, the Ministry of Health and Health Sciences Authority of Singapore jointly issued recommendations for HLA-B*15:02 genotyping before starting carbamazepine (CBZ) in new patients of Asian ancestry as standard of care. The Ministry of Health also approved a 75% subsidy for HLA-B*15:02 genotyping to all patients on subsidy at public healthcare institutions. To understand the impact of these regulatory decisions, we researched the usage patterns for CBZ and levetiracetam, the trend of Stevens–Johnson syndrome/toxic epidermal necrolysis [Stevens–Johnson syndrome (SJS)/toxic epidermal necrolysis (TEN)] reports associated with antiepileptic drugs and the take-up rates of HLA-B*15:02 tests in Singapore. In the 5-year post-policy period, we found that the annual number of reported SJS/TEN cases associated with all antiepileptic drugs was significantly decreased by 57% (p = 0.015); SJS/TEN cases associated with CBZ and phenytoin reduced by 92% and 42% respectively. New CBZ users decreased by 31% while new levetiracetam users approximately doubled. The annual number of HLA-B*15:02 tests conducted increased from 444 to approximately 1,200. Regulatory recommendations for HLA-B*15:02 genotyping as standard of care coupled with government subsidy for the test had contributed to a reduction in CBZ SJS/TEN in Singapore by >90%, in line with that observed in other Asian countries with similar policies. Additionally, the number of phenytoin-SJS/TEN cases also declined. Taken together, this represents a successful example of precision medicine through implementation of a genotyping program to reduce a rare but serious adverse drug reaction among at-risk individuals, while preserving the availability of an effective and low-cost medicine for the broader population.

## Introduction

Carbamazepine (CBZ) is indicated in Singapore for the treatment of epilepsy and other conditions such as diabetic neuropathy, trigeminal neuralgia and bipolar disorders. While CBZ is an effective drug and the drug of choice for several conditions, Stevens–Johnson syndrome (SJS) and toxic epidermal necrolysis (TEN) have been reported with its use. These serious adverse reactions are associated with significant mortality and long-term morbidity. (Pirmohamed et al., 2011).

Published studies had documented a strong association between the carriage of HLA-B*15:02 allele and risk of CBZ-induced SJS/TEN among Han Chinese in Taiwan, Hong Kong, Malays, Indians, and Thais (Chung et al., 2004; Man et al., 2007; Locharernkul et al., 2008; Mehta et al., 2009; Chang et al., 2011). A large prospective study in Taiwan also found HLA-B*15:02 screening prior to the initiation of CBZ therapy to be successful in preventing CBZ-induced SJS/TEN (Chen et al., 2011). Among Han Chinese in Taiwan, HLA-B*15:02 was not associated with CBZ-related drug reaction with eosinophilia and systemic symptoms (DRESS) or maculo-papular erythema (Hung et al., 2006) which are other important phenotypes of severe cutaneous reactions. HLA-B*15:02 also was observed to confer risk to phenytoin SJS–TEN in Han Chinese in Hong Kong and Taiwan, although the association was not as strong as with CBZ (Man et al., 2007; Hung et al., 2010).

Singapore is an island city-state in Southeast Asia with a population of 5.7 million. The three major ethnic groups among the 4.0 million residents are Chinese (74.4%), Malays (13.4%), and Indians (9.0%). The Health Sciences Authority (HSA), in its role as a national pharmacovigilance center, receives spontaneous reports of adverse drug reactions (ADR) related to marketed health products, with the vast majority (94.2%) of cases reported directly by healthcare professionals at public hospitals and primary care clinics. (HSA, 2019). As dermatological reactions comprised the largest category of ADRs received by HSA and local data were deemed necessary to assess the applicability of the HLA-B*1502 association to CBZ-induced SJS/TEN in Singapore, HSA embarked on a program in 2009 to develop infrastructure for collection, storage, and analysis of DNA samples from patients who had experienced serious skin rash, and to capture phenotypic data associated with those samples. For the CBZ-induced SJS/TEN cases collected, all 13 were HLA-B*15:02 positive, as were 3 of the 26 drug-tolerant controls. Hence, the odds ratio (OR) for HLA-B*15:02 association with CBZ SJS–TEN was 181 (95% confidence interval: 8.7–3785, p = 6.9 × 10^-8^), validating a significant association for HLA-B*15:02 in Singapore Chinese and Malays, as has been observed in a number of other Southeast Asian countries. (Toh et al., 2014).

On 30 April 2013, the Singapore Ministry of Health and HSA issued a joint “Dear Healthcare Professional Letter (DHCPL)” advising that genotyping for the HLA-B*15:02 allele before the initiation of CBZ therapy in new patients of Asian ancestry would be standard of care. (HSA, 2013b) The letter further elaborated that “CBZ should not be prescribed prior to the return of HLA-B*15:02 test results” due to the possible development and progression of SJS/TEN in susceptible patients even after prompt discontinuation of the drug. It was advised that patients who were found to be positive for HLA-B*15:02 should not be prescribed CBZ or phenytoin, and treatment alternatives were recommended. Genotyping is not required for patients who have been taking CBZ for three months or longer with no adverse reactions. The Ministry of Health also approved a 75% subsidy for HLA-B*15:02 genotyping to all patients on subsidy at public clinics and hospitals. A few months later, the 2013 CPIC guideline was published advising that CBZ should not be used when it is known that a patient is positive for HLA-B*15:02 (Leckband et al., 2013).

To understand the impact of these regulatory decisions, we conducted this research on the trend of SJS/TEN reports associated with CBZ and other anti-epileptic drugs (AEDs), usage patterns for CBZ, in comparison with levetiracetam (LEV), an alternative AED that is commonly used in Singapore as well as the take-up rates of the HLA-B*15:02 tests in Singapore.

## Methods

### Retrieval of Local SJS and TEN Reports Associated With AEDs

Cases of SJS and TEN associated with the use of CBZ and other AEDs that had reported onset dates between May 2008 and April 2018 were retrieved from HSA's ADR report database and included in the analysis. The AEDs included in this analysis were CBZ, clobazam, gabapentin, lamotrigine, LEV, phenobarbitone, phenytoin, topiramate, and valproate.

### Local Exposure and New Users for CBZ and LEV

Based on consultations with practicing neurologists in Singapore, LEV is the preferred alternative AED. National sales data of CBZ and LEV were used as a proxy for usage of these drugs. Unit sales of all formulations of these products were retrieved from the IQVIA database Singapore National Sales Audit, 2013–2017. The total number of daily defined doses (DDDs) sold annually were calculated, using a DDD of 1.0 g for CBZ and 1.5 g for LEV (WHO, 2019).

The number of new CBZ and LEV users from 2012 to 2017 were tabulated from the Singapore Ministry of Health's prescription database of de-identified prescription orders. Orders for CBZ and LEV issued from 2011 to 2017 were extracted and sorted by order date and pseudo-id. The data were further filtered to retain only the first prescription order tagged to each unique pseudo-id. Thereafter, the number of unique pseudo-ids were sorted by year to tabulate the annual number of new CBZ and LEV users. In order to account for new users who had not been prescribed the drug for at least one year preceding the first order, only data from 2012 onward were used.

### HLA-B*15:02 Genotyping Test

The HLA-B*15:02 genotyping test is offered at four laboratories in Singapore: three public hospital laboratories, namely the National University Hospital Molecular Diagnosis Centre, the Tan Tock Seng Hospital Molecular Diagnostic Laboratory, the DNA Diagnostic & Research Laboratory at Kandang Kerbau Women's and Children's Hospital, and the Tissue Typing Laboratory at Health Sciences Authority. The number of HLA-B*15:02 genotyping tests performed by these laboratories were provided to HSA as part of post-recommendation surveillance. Genotyping was performed by the laboratories using clinically-validated assays developed in-house or using commercially available kits.

### Data Analysis

Descriptive statistics were employed to summarize the data collected, while the Mann–Whitney test was employed to evaluate the differences in the annual number of AED-associated SJS/TEN cases received in the pre- and post-policy periods.

### Ethics Statement

This study was granted approval of exemption by the National Healthcare Group's Domain Specific Review Board which determined that the study qualified for exemption as the research involved analysis of datasets without identifiers.

## Results

### Trends in the Reported SJS–TEN Cases Associated With AEDs

**Figure 1:**
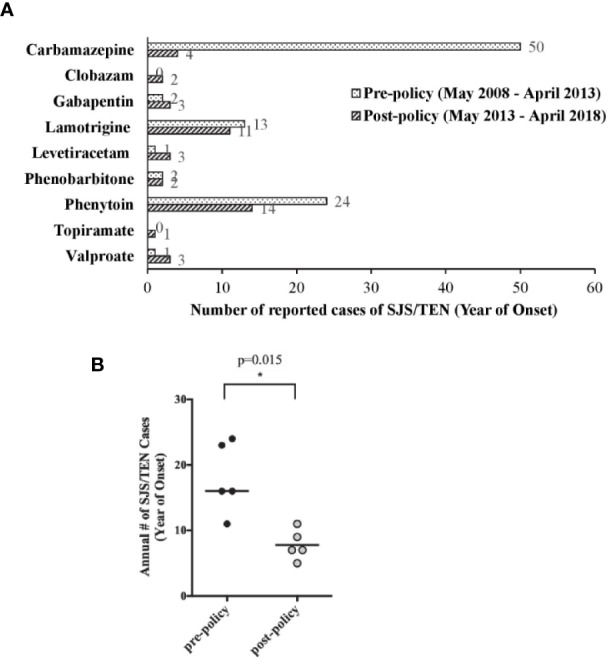
Local spontaneous Stevens–Johnson syndome (SJS)/toxic epidermal necrolysis (TEN) reports associated with anti-epileptic drug (AED) use. **(A)** Comparison of SJS/TEN reports associated with the use of individual AEDs in the pre- and post-policy periods. **(B)** Comparison of annual number of AED-associated SJS/TEN reports in the 5 pre-policy years (May 2008 to April 2013) and 5 post-policy years (May 2013 to April 2018). Horizontal line is the median value, *p = 0.015 by Mann-Whitney test.

The annual number of reported SJS/TEN cases associated with AEDs was significantly decreased by 57% from a 5-year pre-policy period (May 2008 to Apr 2013; median 16, range 11–24) as compared to that in the 5-year post-policy period (May 2013 to Apr 2018; median 7, range 5–11; p = 0.015; **Figure 1A**). In addition, the number of reported cases of SJS/TEN associated with CBZ use decreased sharply by 92% from 50 cases in the pre-policy period to 4 cases in the post-policy period (**Figure 1B**). Genotyping status was reported in 2 cases, of which one was negative for HLA-B*15:02. Moreover, the number of phenytoin-SJS/TEN cases also reduced by 42% from 24 cases to 14 cases in the same time-periods (**Figure 1B**). The numbers of SJS/TEN reports associated with the other AEDs were either stable or slightly increased/decreased, but the numbers were too low for meaningful interpretation.

### Trends in Usage of CBZ and LEV

**Figure 2:**
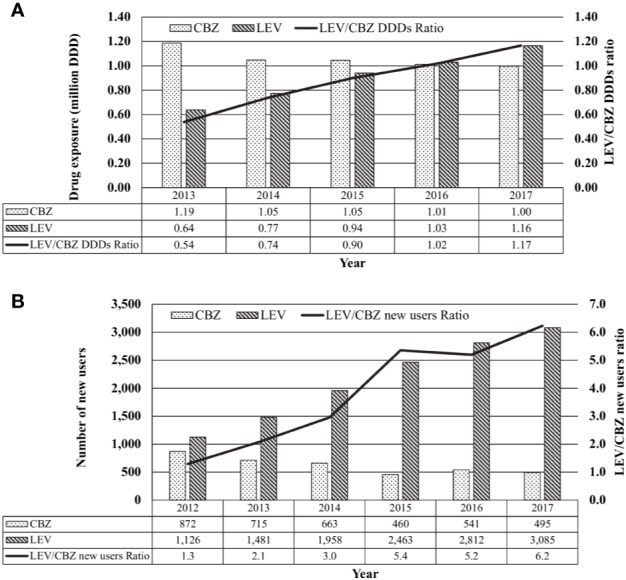
Local usage of carbamazepine (CBZ) and levetiracetam (LEV). **(A)** Total use of CBZ, LEV or CBZ/LEV ratio from 2013 to 2017 based on daily defined dose (DDD), tabulated from IQVIA database Singapore National Sales Audit, 2013–2017. **(B)** New CBZ and LEV users and CBZ/LEV ratio form 2012 to 2017, tabulated from the Singapore Ministry of Health's prescription database.

Between 2013 and 2017, the annual usage of CBZ had decreased slightly by 16% from 1.19 million DDD to 1.00 million DDD while that of LEV increased by 182%, from 0.64 million to 1.16 million DDD (**Figure 2A**).

From 2013 to 2017, the number of new CBZ users in public sector healthcare institutions decreased by 31% from 715 to 495 patients while the number of new LEV users approximately doubled from 1,481 to 3,085 patients (**Figure 2B**).

### Trends in HLA-B*15:02 Screening Rates

**Figure 3:**
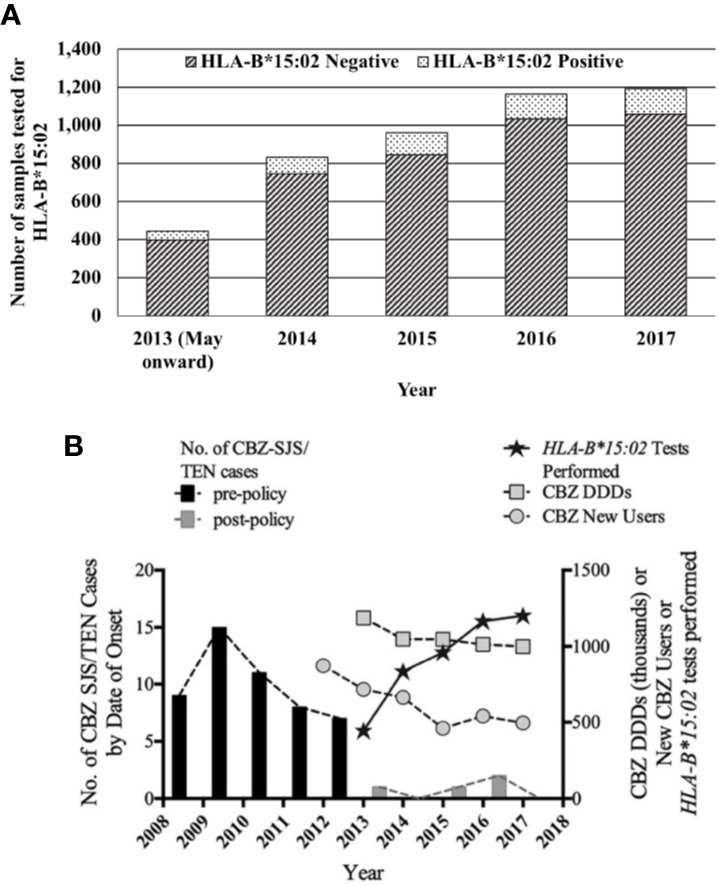
**(A)** HLA-B*15:02 genotyping tests in Singapore in the post-policy period. **(B)** Comparison of CBZ-SJS/TEN cases and trends in genotyping tests. CBZ daily defined doses from national sales data and new users of CBZ from the MOH prescription order database.

From May 2013 to December 2017, a total of 4,595 samples had been sent for HLA-B*15:02 screening at the four laboratories. The number of samples sent per year increased steadily from 444 samples in 2013 to approximately 1,000 cases in 2015, and tapered toward almost 1,200 samples per year in 2016 and 2017. Of all the samples tested for the allele, 11.2% (n = 514) carried the HLA-B*15:02 allele (**Figure 3A**). With the rising number in tests, the number of CBZ-SJS/TEN cases dropped sharply, with only a modest decrease in total sales and new prescriptions of CBZ (**Figure 3B**).

## Discussion

### Impact on SJS/TEN Reports Associated With AEDs

Overall, we observed a significant reduction (57%) in the median annual number of AED-associated SJS/TEN cases received in the post-policy period. This reduction was mainly driven by 92% and 42% reduction in the total number of CBZ- and phenytoin-associated SJS/TEN cases, respectively, during the post-policy period as compared to the pre-policy period. Comparatively, the number of CBZ-associated SJS/TEN cases decreased remarkably by 87.1% in Taiwan (Lin et al., 2018). In Hong Kong, after HLA-B*1502 screening was implemented, the incidence of CBZ-induced SJS/TEN was reduced to zero. However, there was a reciprocal increase in phenytoin-associated SJS/TEN cases in Hong Kong, resulting in non-statistically significant reduction in the overall incidence of AED-associated SJS/TEN after policy implementation (Chen et al., 2014). The authors observed a slight but statistically significant increase (8%) in phenytoin prescription in the post-policy period, and speculated that the policy led to channeling of high-risk patients from CBZ to phenytoin. The changes in the number of SJS/TEN cases associated with AEDs other than CBZ in Taiwan were not discussed in the study by Lin et al. While HLA-B*15:02 is associated with an increased risk of phenytoin–SJS–TEN in Han Chinese, a recent study reports that it is not a risk allele in a Thai population. (Sukasem et al., 2018).

In the Singapore DHCPL issued in April 2013, healthcare professionals were also informed of the suspected association between HLA-B*15:02 and phenytoin-induced SJS/TEN, and advised to consider prescribing drugs other than CBZ and phenytoin for patients tested positive for HLA-B*15:02 allele. This may explain why phenytoin-associated SJS/TEN cases also declined in the post-policy period, unlike the situation in Hong Kong. In addition, as part of our continual effort to maintain healthcare professionals' awareness of the recommendations for HLA-B*15:02 genotyping and early signs of SJS/TEN, we published several articles in the HSA ADR News Bulletin on HLA-B*15:02 genotyping as well as a guide on severe cutaneous adverse reactions and implicated drugs in end–2013 and 2016 (HSA, 2013a; HSA, 2013b; HSA, 2013c; HSA, 2016a; HSA, 2016b). These may have been helpful in reinforcing messaging about genotyping tests and importance of prompt withdrawal of drugs implicated in severe cutaneous skin reactions.

### Impact on Local Usage of CBZ and LEV

Consistent with the findings of Chen et al. and Lin et al., we observed a dramatic decline in the number of new CBZ users during the post-policy period. Nonetheless, when extrapolated to the total usage of CBZ, the decline in new CBZ users was observed to have had minimal impact on the overall usage of CBZ. This could be attributed to continual usage by existing CBZ users who are not affected by the genotyping recommendations and HLA-B*1502 negative patients who are able to use CBZ with very low risk of SJS/TEN. In addition, Chen et al. observed an increase in prescriptions of other AEDs in patients prescribed first-ever AED, with a 3.2-fold increase in the prescription of LEV which was the highest among all the studied AEDs. Likewise, we observed an increasing trend in the number of new LEV users (LEV/CBZ new users ratios of up to 6.2; **Figure 1B**) as well as the overall usage of LEV (LEV/CBZ DDDs ratios of up to 1.2; **Figure 1A**). Notably, the number of new LEV users had begun to increase from 2012 to 2013, prior to the issuance of the local recommendations for pre-treatment HLA-B*15:02 genotyping. LEV is indicated in Singapore as monotherapy and adjunct therapy for the treatment of epilepsy. From our consultation with neurologists, we gathered that LEV had been a favored alternative for epilepsy patients and that the usage of LEV had increased in the recent years. Apart from the availability of generic formulations in recent years, the favorable side effect profile, and ease of use have been cited as reasons for the higher take-up rate for LEV. Moreover, the ability to initiate treatment promptly without the need to wait for pre-treatment genotyping results, had also been seen as a factor favoring its use over CBZ.

### Impact on HLA-B*15:02 Testing

On average, more than 900 tests were ordered per year since 2013. 11.2% of the tests were positive. The proportion of positive tests was in line with the local population HLA-B*15:02 frequency of 11%–18.74% (Dong et al., 2012).

While 4,081 samples were tested negative for HLA-B*15:02 from 2013 to 2017, there were only 2,874 new users of CBZ in the same time period. There could be patients who were not started on CBZ, despite not being found to carry the HLA-B*15:02 allele. Chen et al. and Lin et al. reported that up to 47.2% of patients tested did not have any AED commenced after the test results became available. Another possible reason could be the healthcare professionals' decision to start on other AEDs, instead of CBZ, and continue on the same therapy even after the test results became available, partly due to the inconvenience of added waiting time for the test results. Also, it should be noted that this study was not designed to assess the adherence to HLA-B*15:02 genotyping prior to treatment with CBZ. In Hong Kong and Taiwan, the adherence to HLA-B*15:02 genotyping prior to CBZ therapy was observed in only up to 26.4% of the patients. Further studies are required to assess this issue in the local context.

### Other Considerations

One limitation of our evaluation was the use of different databases for drug sales, drug prescriptions, and genotyping test orders, making it infeasible to trace the intention for genotyping, i.e. whether genotyped patients were those who had intended CBZ use, and the direct impact of these test results on the decision to use CBZ. First-time user data was based on public-sector healthcare system only, and may not be representative of the entire national usage. However, epilepsy is usually treated at specialist and tertiary centers, and approximately 70 to 80% of the overall healthcare demands in Singapore are addressed by the public sector. (Seng et al., 2019). Spontaneous adverse event reporting to HSA is associated with an unknown and variable degree of under-reporting, which is a limitation of our interpretation of SJS/TEN cases reported to this system. In spite of the above, the trend of pre- and post-policy CBZ use in new patients and the reduction in the number of CBZ-associated SJS/TEN cases post-policy were comparable to those observed in other countries that had implemented genetic screening policies. Apart from the number of new users of CBZ and LEV, we were not able to elucidate further information on the characteristics of the new users, such as the patient demographics, the prescribers' medical specialties and the indications for which the medicines were prescribed.

When new clinical care guidelines are published, it often can be difficult to predict the consequences, intended and unintended. When the joint MOH/HSA policy on HLA-B*15:02 genotyping was issued, one concern was that implementation of HLA-B*15:02 genotyping prior to new CBZ use may result in physicians avoiding the use of CBZ, which was considered an effective and low-cost drug of choice for several conditions based on consultations with clinicians. Another concern was that the delay through waiting for the test result would encumber clinical practice and drive physicians to prescribe alternative AEDs. Hence, as a follow-up to issuance of the DHCPL, we have been tracking HLA-B*15:02 test orders and medication usage in addition to the usual pharmacovigilance role of monitoring the number of CBZ-associated SJS/TEN cases. This paper presents the results of that follow-up, namely (1) there has been a >90% decrease in the number of SJS/TEN cases associated with CBZ, (2) HLA-B*15:02 genotyping test orders steadily increased for the first three years and now appears to have reached a steady-state, (3) CBZ continues to be used in clinical practice, albeit at a slightly lower rate, (4) first-time use of CBZ has declined by less than half, and (5) LEV, another AED, has gained in popularity, especially among new users.

## Conclusion

The regulatory recommendations for genotyping for HLA-B*15:02 as “standard-of-care” coupled with government subsidy of 75% for the test has contributed to a reduction in the number of CBZ- and phenytoin-associated SJS/TEN cases in Singapore. CBZ continues to be used in clinical practice though for new AED users, drug utilization of CBZ has decreased while that for LEV has increased. Taken together, this represents a successful example of precision medicine through implementation of a genotyping program to reduce a rare but serious ADR among at-risk individuals, while preserving the availability of an effective and low-cost medicine for the broader population.

## Data Availability Statement

The datasets generated for Figures and statistical analyses are available on request to the corresponding author.

## Author Contributions

CS, DT, and CLC conceived the idea for the research study. CS analyzed the national sales data. ML conducted the data analysis for local ADR reports and HLA-B*15:02 genotyping tests performed. CS and GG retrieved and analyzed data from the Ministry of Health. LT, ML, and CS interpreted the analysis and wrote the manuscript. All authors read and approved the final manuscript.

## Conflict of Interest

The authors declare that the research was conducted in the absence of any commercial or financial relationships that could be construed as a potential conflict of interest.
